# Neuropathogenesis of Japanese Encephalitis in a Primate Model

**DOI:** 10.1371/journal.pntd.0002980

**Published:** 2014-08-07

**Authors:** Khin Saw Aye Myint, Anja Kipar, Richard G. Jarman, Robert V. Gibbons, Guey Chuen Perng, Brian Flanagan, Duangrat Mongkolsirichaikul, Yvonne Van Gessel, Tom Solomon

**Affiliations:** 1 Armed Forces Research Institute of Medical Sciences (AFRIMS), Bangkok, Thailand; 2 Brain Infections Group, Institute of Infection and Global Health, University of Liverpool, NIHR Health Protection Research Unit in Emerging and Zoonotic Infections, and Walton Centre NHS Foundation Trust, Liverpool, United Kingdom; 3 Veterinary Pathology, School of Veterinary Science, and Department of Infection Biology, Institute of Global Health, University of Liverpool, Liverpool, United Kingdom; 4 Emory Vaccine Center, Emory University School of Medicine, Atlanta, Georgia, United States of America; 5 Department of Microbiology and Immunology, College of Medicine, National Cheng Kung University, Tainan, Taiwan; 6 Center of Infectious Disease and Signal Research, National Cheng Kung University, Tainan, Taiwan; 7 Infection Immunology, Department of Clinical Infection, Microbiology and Immunology, Institute of Infection and Global Health, University of Liverpool, Liverpool, United Kingdom; University of Texas Medical Branch, United States of America

## Abstract

**Background:**

Japanese encephalitis (JE) is a major cause of mortality and morbidity for which there is no treatment. In addition to direct viral cytopathology, the inflammatory response is postulated to contribute to the pathogenesis. Our goal was to determine the contribution of bystander effects and inflammatory mediators to neuronal cell death.

**Methodology/Principal Findings:**

Material from a macaque model was used to characterize the inflammatory response and cytopathic effects of JE virus (JEV). Intranasal JEV infection induced a non-suppurative encephalitis, dominated by perivascular, infiltrates of mostly T cells, alongside endothelial cell activation, vascular damage and blood brain barrier (BBB) leakage; in the adjacent parenchyma there was macrophage infiltration, astrocyte and microglia activation. JEV antigen was mostly in neurons, but there was no correlation between intensity of viral infection and degree of inflammatory response. Apoptotic cell death occurred in both infected and non-infected neurons. Interferon-α, which is a microglial activator, was also expressed by both. Tumour Necrosis Factor-α, inducible nitric oxide synthase and nitrotyrosine were expressed by microglial cells, astrocytes and macrophages. The same cells expressed matrix metalloproteinase (MMP)-2 whilst MMP-9 was expressed by neurons.

**Conclusions/Significance:**

The results are consistent with JEV inducing neuronal apoptotic death and release of cytokines that initiate microglial activation and release of pro-inflammatory and apoptotic mediators with subsequent apoptotic death of both infected and uninfected neurons. Activation of astrocytes, microglial and endothelial cells likely contributes to inflammatory cell recruitment and BBB breakdown. It appears that neuronal apoptotic death and activation of microglial cells and astrocytes play a crucial role in the pathogenesis of JE.

## Author Summary

Japanese encephalitis (JE) is one of the most important causes of viral encephalitis worldwide, with no specific antiviral treatment available. Despite some recent successes with widespread vaccination, JE will likely remain an important public health problem; because the virus is mosquito-borne and has natural animal hosts, it will never be eradicated. We have little understanding of what determines the severity and outcome of infection. Data from human post mortem studies is very limited because of cultural constraints on autopsies in areas where JE occurs. Circumstantial evidence suggests that in addition to cytopathology caused directly by infection of neurons, there may be bystander cell death of non-infected neurons, caused by an excessive inflammatory response. Our study used archived brain samples from a prior challenge study in a validated macaque model of JE. We stained for the presence of JEV antigen, apoptosis, and pro-inflammatory markers in affected areas, such as the thalamus and brainstem. We show that bystander neuronal cell death is important, and elucidate the inflammatory and apoptotic mechanisms underlying it. Currently there is no proven efficacious therapy for most viral infections of the central nervous system, including JE. Novel strategies for treating such infections are urgently needed. Our findings suggest new anti-inflammatory and anti-apoptotic therapeutic approaches may be useful in treating this debilitating disease.

## Introduction

Japanese encephalitis virus (JEV) continues to be the leading cause of viral encephalitis in Asia and the Western Pacific, where it is a significant cause of mortality and disability. Annually there are estimated to be up to 70,000 cases, with 10,000–15,000 deaths [1]. Although vaccination is the most viable option to prevent the disease, affordable vaccines are still not widely available, and there is no established treatment for JE.

Despite the disease's importance, little is known about the pathogenesis. During *in vitro* studies neuronal apoptosis was described [2], but its mechanisms and relevance for the disease are still unclear, in particular in relation to the inflammatory response that develops alongside direct viral cytopathology.

Opportunities for in depth neuropathogenic studies on JE in humans are very limited, mainly because autopsy tissue from fatal human cases is rarely available due to cultural constraints in many areas where JE occurs. Mouse models of pathogenesis have some similarities to human disease, but there are also differences [3], [4]. The macaque model, developed in the 1990s to test JE vaccines is a useful model for studying human disease, particularly since the macaque immune system closely resembles that of humans [5]. We therefore conducted a retrospective study on the brains of experimentally JEV-infected macaques, to dissect the inflammatory response and the cascade of events that leads to neuronal damage. We were especially interested in apoptotic pathways and inflammatory mediators including cytokines, inducible nitric oxide synthase (iNOS) and matrix metalloproteinases (MMPs), because these may point towards new targeted treatments to control the inflammatory damage, even in the absence of antiviral therapy.

## Materials and Methods

### Ethics statement

The study does not involve animal use as it was conducted on archived paraffin embedded brain tissue of rhesus macaques (*Macaca mulatta*). The original research on challenge study was conducted in compliance with the Animal Welfare Act and other federal statutes and regulations relating to animals and experiments involving animals and adheres to principles stated in the Guide for the Care and Use of Laboratory Animals, NRC Publication, 1996 edition. The original study was approved by the Institutional Animal Care and Use Committee (United States Army Medical Component, Armed Forces Research Institute of Medical Sciences) and by the Animal Use Review Office, United States Army Medical Research and Materiel Command (Permit Number: 93-11).

### Animals

The study was performed on archived paraffin embedded brain tissue of twelve rhesus macaques challenged intranasally with a well characterized wild-type JEV strain (KE93; Genotype Ia, GenBank accession number KF192510.1) as part of an effort to evaluate second-generation JEV vaccines [5] (Table 1). All archived specimens used in this study are from unvaccinated monkeys. The challenge study had been undertaken in several phases and with different doses, ranging from 7.5×10^5^ to 2×10^10^ plaque forming units [6]. Monkeys originating from India and screened negative for both JEV and Dengue virus neutralizing antibodies (aged 3–7years, of both sexes, weighing 4.0–9.9 kg) had been intranasally inoculated either with the virus isolate passaged twice in culture (animals 1 and 2) or with an isolate prepared from the brain of animal 2 that was subsequently passaged twice in suckling mice to increase both virus titer and virulence [6]. The monkeys were euthanized at the onset of stupor or coma (10–13 days post inoculation) and JEV infection was confirmed by virus isolation from the brain. Five age-matched uninfected control monkeys from an unrelated study served as negative controls.

**Table 1:** Animals, JE challenge virus, infectious doses and time of necropsy.

Animal No.	Sex	Age (yr)	Weight (Kg)	Challenge virus	Challenge dose (pfu)	Day necropsied
1	M	6	6.1	KE93, AP61-1, C6/36-1	2.3×10^7^	12
2	M	7	9.9	KE93, AP61-1, C6/36-1	6.6×10^6^	12
3	M	7	8.5	KE93, AP61-1, C6/36-1, DA-349-1, SM-2	2.0×10^9^	11
4	M	6	4.9	KE93, AP61-1, C6/36-1, DA-349-1, SM-2	2.0×10^9^	11
5	M	5	5.3	KE93, AP61-1, C6/36-1, DA-349-1, SM-2	2.0×10^9^	11
6	M	5	5.2	KE93, AP61-1, C6/36-1, DA-349-1, SM-2	2.0×10^10^	12
7	M	4	4.3	KE93, AP61-1, C6/36-1, DA-349-1, SM-2	2.0×10^10^	10
8	M	3	4.5	KE93, AP61-1, C6/36-1, DA-349-1, SM-2	2.0×10^10^	11
9	M	3	4.0	KE93, AP61-1, C6/36-1, DA-349-1, SM-2	7.5×10^7^	12
10	M	7	9.1	KE93, AP61-1, C6/36-1, DA-349-1, SM-2	7.5×10^7^	10
11	F	7	5.5	KE93, AP61-1, C6/36-1, DA-349-1, SM-2	7.5×10^7^	12
12	F	7	5.6	KE93, AP61-1, C6/36-1, DA-349-1, SM-2	7.5×10^5^	13

pfu – plaque-forming unit.

Animals were euthanized at the onset of stupor or coma.

### Histopathology

Immediately after death, brains were exenterated and sections of frontal lobe, thalamus, brainstem and cerebellum fixed in 10% neutral buffered formalin for at least 72 hours. Following routine paraffin wax embedding, 3–5 µm sections were prepared and stained with haematoxylin-eosin (HE) or used for immunohistology.

### Immunohistology, immunofluorescence and TUNEL method

For immunohistological studies, sections of thalamus and brainstem (exhibiting the most consistent histological changes) and, for comparison, the cortex (absence of inflammatory infiltrates) were chosen. These were stained for the presence of JEV antigen, apoptosis and apoptotic pathway markers, glial and inflammatory cell markers, von Willebrand Factor (to confirm blood brain barrier [BBB] breakdown, through the demonstration of plasma protein leakage), and proinflammatory markers. Commercial antibodies to human proteins were selected for this study, especially those known to cross react with *Macaca mulatta*. Details on the panel of antibodies and the detection methods used are provided in Table S1. Briefly, sections were dewaxed in xylene and hydrated through graded alcohols. To inhibit endogenous peroxidase activity, they were treated with freshly prepared 3% H_2_O_2_ for 15 min. Sections underwent heat-induced antigen/epitope retrieval with a laboratory pressure cooker (Decloaking Chamber, Biocare Medical, Concord, USA) using citrate buffer pH 6 or pH 9 [7]. This was followed by incubation with normal serum to block non-specific binding sites in tissues, and the primary antibodies (15–18 hrs at 4°C) (see Table S1-A). Apoptotic cells were also identified by the terminal deoxynucleotidyl transferase-mediated deoxyuridine triphosphate nick end *in situ* labelling (TUNEL) method using the Apoptag *In Situ* Apoptosis Detection kit (Chemicon Inc., Millipore, Billerica, USA) to demonstrate the characteristic DNA changes. Appropriate controls were included for each marker: uninfected control monkey brains as negative controls for JEV and to establish constitutive expression of other markers, sections with known positivity for specific markers as positive controls, and sections incubated with normal mouse/rabbit IgG as isotype controls.

Double immunolabeling was performed on selected sections of some monkeys (animals 2, 9, 11) to characterize the populations of cells expressing apoptosis markers (TUNEL and caspase-3, -8, and -9) and proinflammatory mediators (cytokines, iNOS and MMPs) and to relate them to the expression of JEV antigen. For this purpose, primary antibodies raised in different species were sequentially localized using non-overlapping secondary reagents and different chromogens (see Table S1-B).

Sequential staining was performed on consecutive sections, mainly to detect tumor necrosis factor alpha (TNF-α) expression in inflammatory cells and glial cells and to further characterize JEV-infected cells when primary antibody were used that had been generated in the same species or when the double immunolabeling was difficult to interpret.

A confocal laser scanning microscope LSM 700 (Carl Zeiss Micro Imaging, Germany) with solid state laser excitation wavelength 488 nm (for FITC) and 555 nm (for Texas Red) and ZEN 2009 software was used to detect immunofluorescent staining. All other light microscopic assessments were undertaken with conventional microscopes.

## Results

### Histopathology and phenotyping of inflammatory response

**Figure 1:**
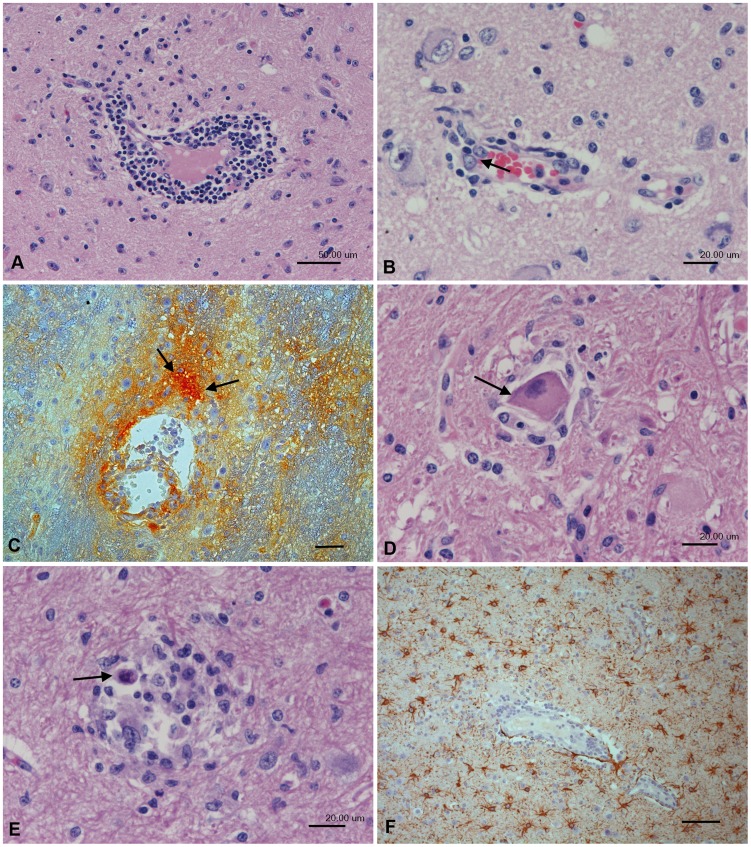
Histopathological changes in the thalamus of a rhesus macaque (No. 2) after intranasal inoculation with JEV. (A) Non-suppurative encephalitis, represented by moderate, lymphocyte-dominated perivascular infiltration. (B) Small vein with mild perivascular infiltration and activated endothelial cells (arrow). (C) The presence of serum, indicated by staining for von Willebrandt factor, in the parenchyma surrounding vessels with perivascular infiltrates (arrows) indicates marked vessel leakage. (D) Degenerating neuron (arrow) surrounded by glial cells (satellitosis). (E) Microglial nodule with occasional apoptotic cells (black arrow). (F) Staining for GFAP highlights the presence of large numbers of activated astrocytes (reactive astrocytosis). A, B, D, E: Hematoxylin-eosin stain. C, F: Indirect peroxidase method, NovaRed (C), DAB (F), hematoxylin counterstain. Scale bars: A, C, F = 50 µm; B, D, E = 20 µm.

**Figure 2:**
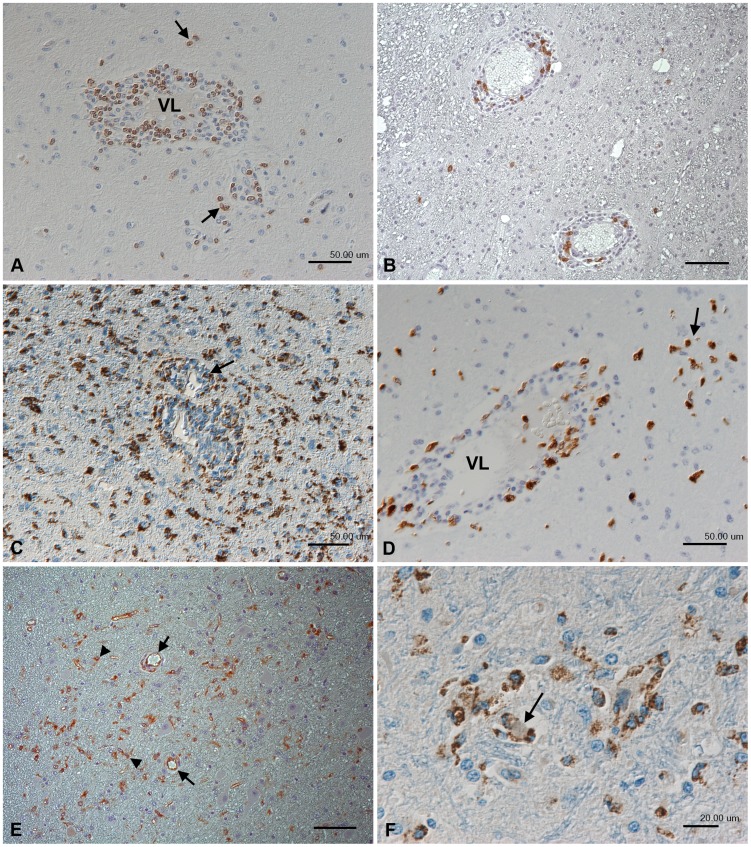
Inflammatory response in the thalamus of rhesus macaques after intranasal inoculation with JEV ((No. 2 (A, B, E) and No. 9 (C, D, F)). (A) CD3+ T cells dominate the perivascular infiltrates and are present in smaller numbers in the adjacent parenchyma (arrows). VL: vessel lumen. (B) CD20+ B cells represent a minority in the perivascular infiltrates. (C) Staining for CD68 identifies moderate numbers of macrophage/microglial cells within and surrounding the perivascular infiltrates (arrows) and highlights the large number of disseminated activated microglial cells in the adjacent parenchyma. (D) Macrophages in the perivascular infiltrates and the adjacent parenchyma (arrow) also express the myeloid/histiocyte antigen which indicates that they have recently been recruited from the blood. VL: vessel lumen. (E) Activated microglial cells also express major histocompatibility complex (MHC) class II antigen (arrowheads). MHC II is also expressed by vascular endothelial cells (arrows), confirming their activation. (F) Microglial nodule with central degenerate neuron (arrow), surrounded by CD68-positive microglial cells. Indirect peroxidise method, DAB, Papanicolaou's hematoxylin counterstain. Scale bars: A–E = 50 µm; F = 20 µm.

All JEV-infected animals exhibited mild to moderate, multifocal to diffuse, non-suppurative meningoencephalomyelitis with evidence of neuronal degeneration and death. The inflammatory response was similar in its extent and composition regardless of the dose of inoculum and the day of euthanasia, and was dominated by mononuclear perivascular cuffs (Figure 1A) and meningeal infiltrates. These were accompanied by morphological evidence of endothelial cell activation (represented by a tomb-stone like luminal protrusion of endothelial cells; Figure 1B) and/or vascular damage. The latter was indicated by perivascular haemorrhage and substantial leakage of serum into the parenchyma, as demonstrated by staining for von Willebrand factor (Figure 1C). Neuronal cell death was indicated by morphological neuronal changes suggestive of apoptosis, in association with satellitosis or microglial nodules (Figure 1D,E). Reactive astrogliosis, represented by a multifocal increase in astrocyte numbers (Figure 1F) and evidence of astrocyte activation (presence of gemistocytes) in areas with inflammatory infiltrates was also identified in JEV infective brains. T cells (CD3+) were the predominant leukocytes in both perivascular and meningeal infiltrates. They were also present in small numbers in the adjacent parenchyma (Figure 2A). B cells (CD20+) were sparse and primarily seen in the perivascular infiltrates (Figure 2B), while moderate numbers of macrophage/microglial cells (CD68+) identified in perivascular and meningeal infiltrates and the adjacent brain parenchyma (Figure 2C). Staining for myeloid/histiocyte antigen, reported to stain macrophages [8] and microglial cells [9], identified a substantial number of cells with a morphological appearance of macrophages (Figure 2D), suggesting their recruitment into the tissue. Staining for CD68, which is also expressed by microglial cells, and major histocompatibility complex (MHC) class II antigen (expressed mainly by activated microglial cells) confirmed the presence of microglial nodules but also demonstrated diffuse microgliosis and activation of microglial cells (presence of both reactive and amoeboid microglial cells; Figure 2 C,E). Furthermore, endothelial cells were shown to express MHC II, confirming their activation (Figure 2E). The cells surrounding neurons in satellitosis were also CD68-positive microglial cells (Figure 2F). For comparison, in brain areas without evidence of viral antigen and inflammation (cerebral cortex), only scattered MHCII-positive microglial cells without morphological features of activation were seen. There was no evidence of microglial MHC II expression in control brains.

### Identification of JEV target cells

**Figure 3:**
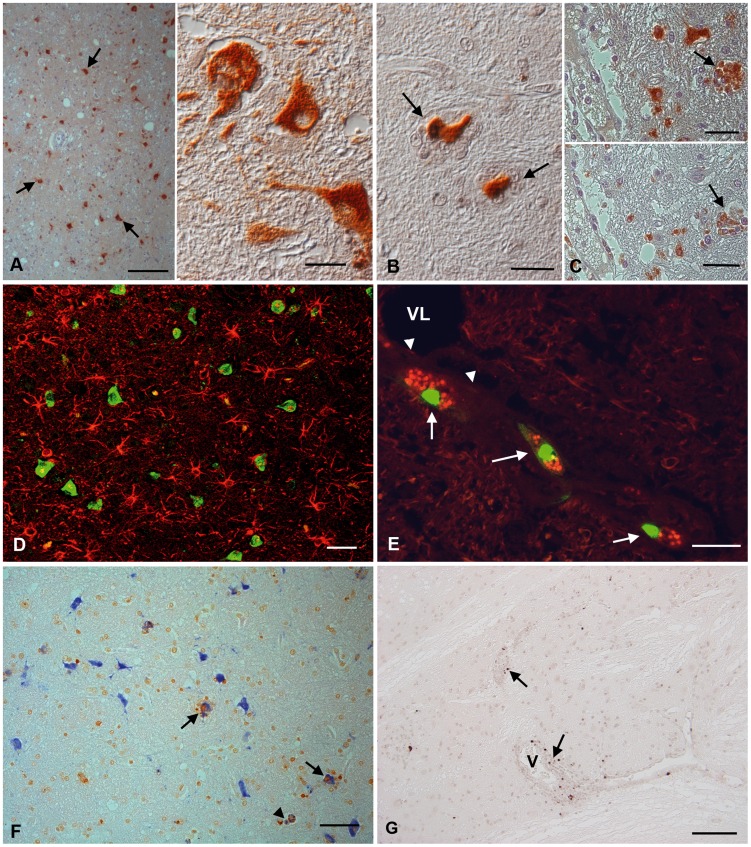
JEV target cells in the thalamus of rhesus macaques after intranasal inoculation with JEV ((No. 7 (A, B), No. 2 (C–G)). (A) JEV antigen is seen in the majority of neurons (left: arrows). Right: Infected unaltered neurons express viral antigen in both cell body and cell processes. (B) JEV-infected neurons that are surrounded by microglial cells in satellitosis appear shrunken (arrows). (C) Microglial cells in particular in microglial nodules can be JEV-infected (top; arrow) and are identified based on their CD68 expression (bottom; arrow), as demonstrated in a consecutive section. (D) Dual staining for JEV antigen (FITC) and GFAP (Texas red) indicates that JEV does not infect astrocytes. (E) While endothelial cells (arrowheads) were not found to be JEV infected, perivascular macrophages in one animal were found to express JEV antigen (Texas Red); these cells were also undergoing apoptosis, since they were TUNEL-positive (FITC) (arrows). VL: vessel lumen. (F) Dual staining for JEV antigen (Vector Blue) and TUNEL (DAB) shows both the degenerating neurons and surrounding microglial cells in satellitosis undergo apoptosis (arrows). JEV-infected, apoptotic microglial cells (arrowhead) are also observed. (G) Occasional TUNEL-positive, apoptotic lymphocytes (arrows) are present in the perivascular infiltrates. V: vessel. Indirect peroxidase method (A–E, G), Vectastain Elite ABC-Alkaline Phosphatase Kit (F). DAB (A–G), *BCIP*/NBT blue (F), Papanicolaou's hematoxylin counterstain. Scale bars: A (left) = 100 µm; A (right), C = 25 µm; B, E = 20 µm; D, F, G = 50 µm.

JEV antigen expression, seen as finely granular cytoplasmic staining, was observed in numerous neuronal cell bodies and processes disseminated in the thalamic and brain stem nuclei of all animals and in neuronal cell processes throughout the affected parenchyma (Figure 3A). Most infected neurons appeared morphologically unaltered (Fig. 3A inset), but some were surrounded by microglial cells (satellitosis) and exhibited degenerative changes (Figure 3B). JEV-positive microglial cells were found in some glial nodules, but occasionally as individual cells in affected areas like brainstem and thalamus, as confirmed by sequential staining for CD68 and JEV antigen (Figure 3C). In contrast, there was no evidence of JEV infection of astrocytes (Figure 3D). In one animal with a particularly strong inflammatory response (animal 2), a small percentage of slender perivascular cells (perivascular macrophages) also expressed viral antigen (Figure 3E). There was no evidence of JEV antigen in endothelial cells in any animal. Nor was there any correlation between intensity of viral infection as indicated by immunostaining and degree of inflammatory response. Negative control brain sections did not show any positive reaction.

### Apoptosis

Morphological features of apoptosis were observed in degenerating neurons within glial nodules and in satellitosis, among leukocytes in the perivascular infiltrates and in individual cells with microglial features in the adjacent parenchyma. Cell death by apoptosis was confirmed by the TUNEL method which identified apoptotic JEV-infected neurons in glial nodules and satellitosis as well as apoptotic microglial cells disseminated in the parenchyma, in satellitosis and in microglial nodules (Figure 3F). Occasional lymphocytes in the perivascular infiltrates were also apoptotic (Figure 3G) and the JEV-infected perivascular macrophages were apoptotic in animal 2 (Figure 3E).

**Figure 4:**
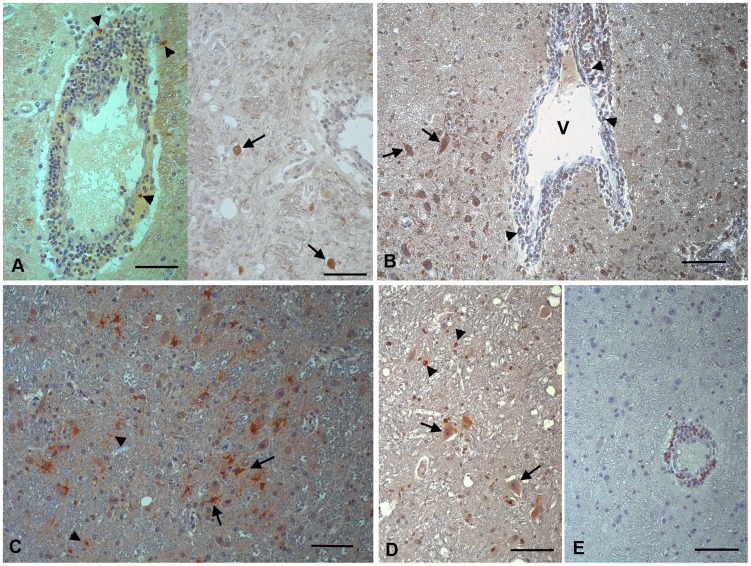
Apoptosis related proteins in the thalamus of rhesus macaques after intranasal inoculation with JEV ((No. 2 (A, D, E), No. 9 (B), No. 11 (C)). (A) Some leukocytes in the perivascular infiltrates (left, arrowheads) and scattered unaltered appearing neurons (right; arrows) express cleaved caspase-3, an executor caspase. (B) The initiator caspase-8 is expressed by unaltered neurons (arrows) and some cells in the perivascular infiltrates (arrowheads). V: vessel. (C) Caspase-9, another initiator caspase, is expressed by microglial cells (arrowheads) and astrocytes (arrows). (D) Bax, a pro-apoptotic protein, is expressed by unaltered neurons (arrows) and microglial cells (arrowheads). (E) Bcl-2, an anti-apoptotic protein, is expressed by cells in the perivascular infiltrates. Indirect peroxidise method, DAB, Papanicolaou's hematoxylin counterstain. Scale bars = 50 µm.

Key apoptosis molecules, including caspases-8, -9 (both initiator caspases) and cleaved caspase-3 (an executor caspase) were identified by staining to detect cells undergoing early apoptosis and not exhibiting representative morphological features. Small numbers of neurons with normal morphology expressing cleaved caspase-3 and more cells expressing caspase-8 were seen in JEV infected brains. Both caspases were also expressed by some leukocytes in the perivascular infiltrates (Figure 4A, B). Caspase-9, however, was only detected in astrocytes and microglial cells (Figure 4C). Double staining for JEV and the various apoptosis markers confirmed that some JEV-infected neurons were undergoing apoptosis (data not shown).

In order to better understand the regulation of apoptotic processes in response to JEV infection, the expression of representative pro- and anti-apoptotic proteins was assessed. While numerous microglial cells and occasional neurons stained positive for the pro-apoptotic protein Bax (Figure 4D), the anti-apoptotic protein Bcl-2 was mainly expressed by lymphocytes in the perivascular infiltrates (Figure 4E).Dual staining showed JEV antigen in some Bax-positive neurons and occasional Bax-positive microglial cells (data not shown).

In uninfected control brains TUNEL positive cells were not identified. Caspase and Bcl-2 staining was negligible; weak and infrequent Bax expression was seen in neurons.

### Proinflammatory mediators

**Figure 5:**
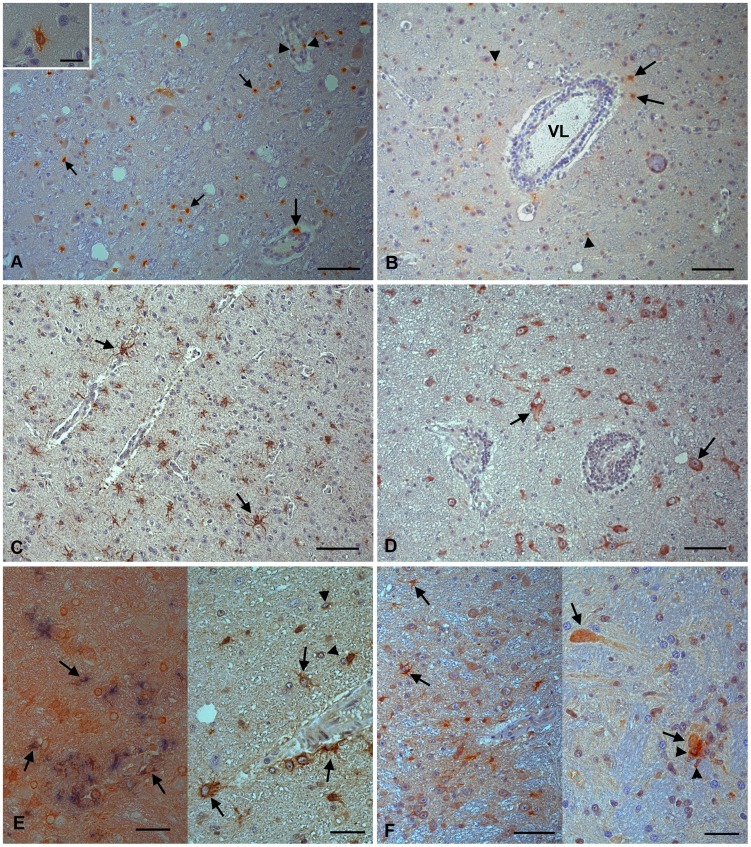
Proinflammatory markers in the thalamus of rhesus macaques after intranasal inoculation with JEV (No. 2 (A, B, D–F), No. 11 (C)). (A) Microglial cells (small arrows), leukocytes in the perivascular infiltrates (arrowheads), perivascular macrophages (large arrow) and astrocytes (inset) express iNOS. (B) Nitrotyrosine expression is observed in microglial cells (arrowheads) and astrocytes (arrows). VL: vessel lumen. (C) MMP-2 expression is diffusely seen in reactive astrocytes. (D) MMP-9 is mainly expressed by neurons. (E) TNF-α (left: brown signal) is expressed by microglial cells (left: arrows; right: arrowheads) that are identified based on their CD68 expression (left: blue signal) and astrocytes (right: arrows). (F) IFN-α expression is seen in astrocytes (left; arrow) and neurons, both unaltered (left: arrowheads; right: arrow) and degenerating (right: arrowhead), as demonstrated in satellitosis. Microglial cells surrounding the neuron are also positive. Indirect peroxidase method (A–F), Vectastain Elite ABC-Alkaline Phosphatase Kit (E, left); DAB (A–F), *BCIP*/NBT blue (E, left), Papanicolaou's hematoxylin counterstain. Scale bars A–D, F left = 50 µm. E, F right = 20 µm.

Having characterized the inflammatory response and the patterns of cell death in the brains for monkeys infected with JEV, we aimed to identify relevant mediators of these processes frequently identified in viral mediated infections. To assess local nitric oxide (NO) production, we investigated the expression of iNOS and nitrotyrosine (NT). We stained for MMP-2 and -9, which are known to cause BBB disruption by degrading collagen IV, its main component [10], interferon (IFN)-α, a potent antiviral cytokine and microglial activator [11], and TNF-α which has been shown to directly activate microglia [12] and induce neuronal apoptosis [13]. Both iNOS and NT were expressed by microglial cells and astrocytes. iNOS expression was also seen in some macrophages in the perivascular infiltrates and the adjacent parenchyma (Figure 5A,B) where staining for NT was only very weak. MMP-2 was expressed in cells with the morphology of reactive astrocytes (Figure 5C) and, to a lesser extent, in microglial cells and in infiltrating macrophages, whereas MMP-9, known to be constitutively expressed in human neurons, was intensely expressed by neurons and relatively weakly by microglial cells (Figure 5D). TNF-α expression was seen in microglial cells, infiltrating macrophages and astrocytes, as confirmed by dual staining with CD68 and sequential staining with GFAP (Figure 5E). It was also occasionally seen in endothelial cells (data not shown). IFN-α expression, however, was seen both in uninfected and infected neurons, as confirmed by dual staining with JEV antigen (data not shown), and in astrocytes and microglial cells (Figure 5F). In control brains, only minimal expression of inflammatory mediators was seen, represented by staining in occasional vascular endothelial cells (iNOS, TNF-α), neurons (MMP-9, iNOS) and vascular smooth muscle cells (TNF-α).

## Discussion

The present study used macaques, which have previously been established as a good model for neuropathological studies on JE in humans [5], [6], to evaluate the cytopathic effects of and inflammatory response to JEV in the brain. The apoptosis pathways and the full spectrum of proinflammatory factors have not been fully studied in any previous animal models of JE, or autopsy tissues. This study utilized monkeys challenged with JEV intranasally rather than a route more consistent to natural infections to increase the likelihood of encephalitis. Peripherally challenged monkeys generally do not typically develop encephalitis [14] and with direct intracerebral challenge the encephalitis develops early [15]. The intranasal route was therefore the most useful route in our model and has been reported to provide a useful model for the study of anti-viral compounds and vaccine candidates [5], [15] albeit this unnatural infection route may be a limitation in our study.

As in humans, JEV induces a non-suppurative meningoencephalitis with neuronal cell death, microgliosis and astrogliosis in macaques [16], [17]; these classic findings are also common in other viral encephalitides [18]. However, the ‘punched-out’ areas of focal necrosis, often seen in fatal human JE cases [16], [19] were not observed in our experimentally infected monkeys. It is possible that this pathology had not yet developed in the macaques that were euthanized at the onset of stupor or coma in contrast to human infections where histological observations are always made on post mortem material at the end of the disease process [16], [19].

The inflammatory response in macaques even with the chosen challenge route was consistent with the changes seen in humans, characterised by perivascular mononuclear cuffs, with less intense infiltrates in the adjacent parenchyma [16]. While T cells dominated in the perivascular infiltrates and recently recruited macrophages were the largest population in the parenchymal infiltrates, B cells represented a minority and were restricted to the perivascular cuffs. Cytotoxic T cells (CTLs) have been reported to play a key role in mouse models of JE [20], but it remains unclear if these cells are beneficial or deleterious, or both. In the present study, it was not possible to assess the role of CTLs, due to the non-availability of antibodies suitable for macaques. In viral encephalitis, macrophages are known to migrate from the perivascular space into the surrounding parenchyma where they become activated [21].In addition to microglia, known to cause neuronal death in JE [3], [19], the relative contribution of peripheral macrophages that migrate into the CNS should be elucidated.

Our study confirmed neurons as the main targets of JEV, as previously shown in fatal human cases [16], [19], [22]. We also demonstrated viral antigen in microglial cells, mainly within microglial nodules surrounding infected neurons, suggesting virus uptake by phagocytosis. However, productively infected microglial cells cannot be excluded, since they do support viral replication *in vitro* [23], [24]. Viral antigen was not detected in other glial cell types, despite evidence that astrocytes can become infected in culture systems [23]. There was also no evidence of endothelial cell infection. A similar viral target cell pattern has been reported in human cases, with the exception that some studies found evidence also for endothelial cell infection [16], [19]. Interestingly, we detected JEV antigen in perivascular macrophages in one animal. These cells found at the interface between blood and brain parenchyma are resident macrophages with high phagocytic activity and MHC-II expression [25], which suggests that they had phagocytosed virus that entered the brain via the blood.

Viral infection and inflammatory responses were associated with cytopathic changes, and, although not excessive, neuronal death via apoptosis was clearly observed. Apoptosis was shown by the TUNEL assay which has been used in the past to demonstrate apoptosis, although interpretation of the findings can be difficult in the presence of necrosis and autolytic changes [26]; we therefore also confirmed apoptosis by staining for cleaved caspase-3. Apoptotic neurons were often surrounded by microglial cells (satellitosis and formation of microglial nodules) which indicated their impending phagocytosis. Some apoptotic neurons were JEV infected. In addition, several morphologically unaltered, infected neurons were shown to express the pro-apoptotic protein Bax, the initiator caspase-8 or the active effector caspase-3, which indicates that these cells were destined to become apoptotic. These results confirm the *in vivo* relevance of previous *in vitro* studies which demonstrated that JEV replication can lead to neuronal apoptotic death [27] and support findings from the mouse model that JEV replication contributes to Bax activation [28]. Taken together, these findings provide clear evidence of a direct, although possibly not rapid, cytopathic effect of JEV on neurons. The demonstration of caspase-8 in affected neurons also indicates that neuronal apoptosis is initiated by the fas-mediated or extrinsic pathway, a mechanism that is central to the process of immune-mediated viral clearance [29] and seen in a number of CNS viral infections including West Nile virus [30].

Importantly, apoptotic cell death or pre-apoptotic caspase-8 expression was also seen in a proportion of JEV antigen-negative neurons, which suggests some degree of bystander neuronal death. In addition, a proportion of microglial cells, often in close proximity to infected neurons but generally not JEV-infected, were apoptotic. Furthermore, the observation of morphologically unaltered microglial cells expressing caspase-9 suggest that microglial apoptosis is initiated by the mitochondria or the intrinsic pathway. A recent *in vitro* study showed that JEV infection can lead to apoptosis of microglial cells [24].Our results indicate that *in vivo* this direct mechanism is probably less relevant and that pro-inflammatory factors are more important; this is also seen in other CNS conditions, such as experimental autoimmune encephalomyelitis (EAE) where microglial apoptosis is considered an important homeostatic mechanism to control microglial activation and proliferation [31]. Apoptotic cell death was also observed in a proportion of infiltrating inflammatory cells in our JEV infected monkeys. Considering that these cells were not JEV-infected, this most likely represents a normal mechanism to eliminate activated leukocytes and thereby limit the inflammatory response in the CNS. On the other hand, infiltrating leukocytes (predominantly T cells) were found to express the anti-apoptotic protein Bcl-2. This supports a murine *in vivo* study that provides evidence of a critical role of Bcl-2 in the survival of virus-specific CTLs [32].

The occurrence of apoptosis in apparently uninfected neurons suggests that indirect mechanisms (bystander cell death) contribute to neuronal damage in JE, and indeed recent *in vitro* and *in vivo* murine studies demonstrated that microglial cells can induce neuronal apoptosis via the release of pro-inflammatory mediators [3], [4]. Also, TNF-α, via its receptor on neurons, has been shown to induce caspase-8 activation in mouse neurons [33]. Indeed, we observed TNF-α upregulation in astrocytes, microglial cells, endothelial cells and infiltrating macrophages in infected macaques. It is likely that these cells were also responsible for the TNF-α upregulation observed in JEV-infected mice [3], [34]. TNF-α related neuronal death is also reported in a recent *in vitro* study with WNV [35]. The results of our study suggest that JEV might simultaneously trigger, both directly and indirectly, the caspase dependent extrinsic apoptotic pathway in neurons and the intrinsic apoptotic pathway in microglial cells. Further definition of the underlying mechanisms will allow us to understand the processes involved in disease progression and to assess the potential of anti-apoptotic treatment strategies.

Alongside the inflammatory infiltration and the cytopathic effects, we found distinct evidence of activation of a range of cells, namely microglial cells, astrocytes and vascular endothelial cells. Microglial activation was confirmed by the demonstration of MHC II antigen, iNOS, NT, TNF-α and MMP expression by microglial cells and has been reported previously in JEV-infected mice [3]. To shed light on the potential mechanism of microglial activation, we assessed the expression of IFN-α (type I IFN); this potent antiviral cytokine is an activator of microglia in response to CNS viral infection [11], and is elevated in the cerebrospinal fluid of patients with JE, where it is associated with a poor outcome [36]. We demonstrated IFN-α expression in neurons which suggests that they might be responsible for microglial activation early after infection; expression by microglia and astrocytes suggests they might be responsible for sustained microglial activation in JE.

As described in earlier reports [22], reactive astrogliosis and astrocyte activation was also observed in the present study. Astrocyte activation is considered as a non-specific response to degenerative changes including virus-induced damage in the CNS. However, a recent study provided evidence that this activation might be an effect of TNF-α release from microglial cells [23]. So far, little is known about the role of astrocytes in neuroinflammation caused by JEV, whether they are protective or pathogenic. Nevertheless, the demonstration of TNF-α, IFN-α, iNOS, NT and MMP-2 expression by astrocytes in our study provides the first *in vivo* evidence that astrocytes may play an important role in the pathogenesis. The same is true for microglial cells and macrophages in the inflammatory infiltrates, through release of the inflammatory mediators, all these cells might actively contribute to the damage of other cells in the brain and in particular induce bystander apoptotic death of neurons [3], [4]. iNOS and NT expression indicate NO production, which is in accordance with results from a mouse study [37]. There, a gradual increase in iNOS activity was observed after intracranial JEV infection, and was considered a consequence of release of cytokines, such as TNF-α or IL-8 which might be beneficial through the inhibition of viral replication and release [37]. However, NO has also been discussed as a potential mediator of pathogenesis in tick-borne encephalitis virus infection [38]. MMP levels have been shown to correlate with the severity of some CNS infections [39]. MMP-9 is known to be constitutively expressed in human neurons. However, it was intensely upregulated in neurons of the JEV-infected macaques and weakly expressed by microglial cells, while glial cells and infiltrating macrophages were sources of MMP-2. MMP release is stimulated by proinflammatory cytokines including TNF-α [40]. In JE, MMPs might play a detrimental role and not only be responsible for BBB disruption through collagen IV degradation, but also contribute to neuronal destruction via stimulation of TNF-α release.

We observed endothelial cell expression of MHC II antigen and TNF-α, which confirms that they are activated and suggests they have a role in inflammatory cell recruitment and potential contribution to immune reactions, glial cell activation and neuronal apoptosis. Endothelial cells might also be a source of the increase in serum TNF-α seen in JE patients [36].

Based on our findings we postulate that infection of neurons by JEV triggers a network of inflammatory mediators [41]. Through release of IFN-α, neurons activate microglial cells which, via release of cytokines such as TNF-α, activate astrocytes and endothelial cells. Together, these mediators contribute to BBB breakdown, leukocyte recruitment into the parenchyma and further neuronal apoptosis. Glial cell apoptosis should limit the extent of inflammation. However, the release of further mediators by infiltrating leukocytes, in particular macrophages, results in sustained glial and endothelial cell activation and further leukocyte recruitment, ultimately augmenting the inflammatory response and neuronal cell loss. Although the inflammatory response is intended to be protective, and presumably is so in cases which improve and recover, if uncontrolled it can contribute to disease progression in JE.

Our study is mostly descriptive as we used archived materials from a previous challenge study. However it might shed some light on some novel processes mediating pathogenesis which could aid in the experimental design for future studies investigating inflammatory responses to JE. Viral encephalitis is a major cause of morbidity and mortality worldwide. The pathogenesis of flavivirus encephalitis remains incompletely understood but it appears that the immune response is crucial in limiting viral spread to the brain [42]. The cascade of events that we have outlined for JE may also apply to other viral encephalitides. Currently there is no proven efficacious therapy for most viral infections of the CNS including JE. Novel strategies for treating viral CNS infections are urgently needed. Our results from a macaque model indicate that neuronal apoptosis and glial activation are crucial steps in the pathogenesis of JE. They imply that adjunctive therapy with inhibitors of caspases or targeted anti-inflammatory treatments might be a promising therapeutic approach for JE in the future.

## Supporting Information

Table S1 Immunostaining of Japanese encephalitis virus infected monkey brains.
(PDF)
