# Supplementary material for: Neuropathogenesis of Japanese Encephalitis in a Primate Model
Source: PLoS Negl Trop Dis. 2014 Aug 7;8(8):e2980. doi: 10.1371/journal.pntd.0002980 (PMC4125110; doi:10.1371/journal.pntd.0002980)
Supplement: Table S1 — Immunostaining of Japanese encephalitis virus infected monkey brains. (PDF) [file pntd.0002980.s001.pdf]

Supplemental Material Table S1. Immunostaining of Japanese Encephalitis Virus infected monkey brains.

A. Antibodies and detection methods.

Antibody (clone)	Source	Detection method*	Dilution	Specificity
Mouse anti-JEV	AFRIMS	IP&IFA	1:2000/1:160	Japanese encephalitis virus
Mouse anti-Neuronal Nuclei [NeuN]	Chemicon	IP	1:200	Neurons
Rabbit anti-Glial Fibrillary Acidic Protein [GFAP]	DakoCytomation	IP&IFA	1:1000/1:100	Astrocytes
Anti-Human Von Willebrand Factor [Factor VIII]	DakoCytomation	IP	1:1600	Endothelial cells, megakaryocytes, platelets, serum
Rabbit anti-Human CD3	DakoCytomation	IP	1:100	T cells
Mouse anti –Human CD20 cy (L26)	DakoCytomation	IP	1:200	B cells
Mouse anti –Human CD68 (KP1)	DakoCytomation	IP	1:100	Macrophages, monocytes, microglial cells
Mouse anti-Human Myeloid/Histiocyte Antigen (MAC 387)	DakoCytomation	IP	1:1600	Monocytes, neutrophils, recently blood derived macrophages, some microglial cells (8,9)
Mouse anti-Human HLA-DR Antigen [MHC II] (TAL.1B5)	DakoCytomation	IP	1:25	Macrophages, B cells, activated T cells, activated microglial cells, activated endothelial cells
Rabbit anti-Human/Mouse Cleaved Caspase-3 [Asp175]	R & D	IP	1:100	Cleaved Caspase-3

Rabbit Caspase-8 p18 [H-134])	Santa Cruz	IP	1:50	Caspase-8
Rabbit Caspase-9 p10 [H-83])	Santa Cruz	IP	1:50	Caspase-9
Rabbit anti-Human BAX [N-20]	Santa Cruz	IP	1:2000	Baxα, Baxβ, Bax
Mouse anti-Human Bcl-2 (100/D5)	Novocastra	IP	1:80	Human bcl-2 oncoprotein
Rabbit anti-Nitric Oxide Synthase [iNOS]	Thermo Scientific	IP	1:150	Activated macrophages and glial cells
Rabbit anti-Nitrotyrosine [NT]	Chemicon	IP	1:200	Nitrotyrosine
Mouse anti-Human Matrix Metalloproteinase [MMP]-2 (A-GelVC2)	Neo Marker	IP	1:10	Both pro and active forms of MMP-2
Rabbit anti-Human Matrix Metalloproteinase [MMP]-9	Neo Marker	IP	1:750	Both pro and active forms of MMP-9
Rabbit anti-Human Tumor Necrosis Factor [TNF]-α	AbCam	IP	1:1000	Cell bound pre-cursor of TNF-α
Rabbit anti-Human Interferon [IFN]-α	PBL Biomedical Laboratories	IP	1:2000	IFN-α

IP = Indirect peroxidase with peroxidase-conjugated anti-mouse or -rabbit antibody (EnVision Systems; Dako, Carpinteria, CA, USA) and diaminobenzindine (DAB, VECTOR, Burlingame, CA, USA), NovaRED (VECTOR) or tetramethylbenzidine (TMB, VECTOR) as substrate

IFA = Indirect immunofluorescence with goat anti-rabbit FITC (kpl, Gaithersburg, MD, USA), sheep anti-mouse IgG FITC conjugate (Sigma,

St. Louis, MO, USA), goat anti-rabbit Texas Red (Molecular Probes, Eugene, OR, USA) or goat anti-mouse Texas Red (Molecular Probes).

*pre-treated with heat-induced antigen/epitope retrieval (HIER) in citrate buffer pH 6.0 (VECTOR) for all antigens except for Bcl-2 (citrate buffer pH 9.0

Sources:

AFRIMS, Armed Forces Research Institute of Medical Sciences, Bangkok, Thailand

Chemicon International, Inc, Temecula, CA, USA

DykoCytomation, Carpinteria, CA, USA

R&D, Minneapolis, MN, USA

Santa Cruz Biotecnology, Santa Cruz, CA, USA

Novocastra, New Castle, UK

Thermo Scientific, Rockford, IL, USA

Neo Marker, Fremont, CA, USA

AbCam, Cambridge, UK

PBL Biomedical Laboratories, Piscataway, NJ, USA

B. Double and sequential immunostaining of Japanese Encephalitis Virus infected monkey brains

Antibody		Detection Method (chromogen/fluorochrome)	
First	Second	First	Second
Double Immunostainng			
TUNEL	JEV	IFA/FITC	IFA/TR
		IP/DAB	ABC/AP
	NeuN	IP/DAB	ABC/AP
	CD68	IP/DAB	ABC/AP
	MAC387	IP/DAB	ABC/AP
JEV	GFAP	IFA/FITC	IFA/TR
		ABC/AP	IP/DAB
	IFN-α	ABC/AP	IP/DAB
	TNF-α	ABC/AP	IP/DAB
	MMP-9	ABC/AP	IP/DAB
	iNOS	ABC/AP	IP/DAB
	Cleaved Caspase-3	ABC/AP	IP/DAB
	Caspase-8	ABC/AP	IP/DAB
	Caspase-9	ABC/AP	IP/DAB
	BAX	ABC/AP	IP/DAB
CD68	iNOS	ABC/AP	IP/DAB

	MMP-9	ABC/AP	IP/DAB
	TNF-α	ABC/AP	IP/DAB
Sequential Immunostaining			
JEV	CD68	IP/DAB	IP/DAB
TNF-α	GFAP	IP/DAB	IP/DAB
	CD3	IP/DAB	IP/DAB
	CD68	IP/DAB	IP/DAB

IP/DAB = Indirect peroxidase with peroxidase-conjugated anti-mouse or -rabbit antibody (EnVision Systems; Dako, Carpinteria, CA) and diaminobenzindine (DAB, VECTOR, Burlingame, CA, USA) or NovaRED (VECTOR) as substrate

ABC/AP = Avidin Biotin Complex – Alkaline Phosphatase with 5-bromo-4-chloro-3-indolyl phosphate/nitroblue tetrazolium (VECTOR Blue) as substrate

IFA/FITC = Indirect immunofluorescence with goat anti-rabbit FITC (kpl, Gaithersburg, MD, USA) or sheep anti-mouse IgG FITC conjugate (Sigma, St. Louis, MO, USA)

IFA/TR = Indirect immunofluorescence with goat anti-rabbit Texas Red (Molecular Probes, Eugene, OR, USA) or goat anti-mouse Texas Red (Molecular Probes).
